# “Invisibilidad” como espejo del malestar civilizatorio: el aumento de la población en situación de calle en Brasil

**DOI:** 10.18294/sc.2025.6039

**Published:** 2025-12-10

**Authors:** Sonia Fleury

**Affiliations:** 1 Doctora en Ciencias Políticas. Investigadora sénior, Centro de Estudos Estratégicos, Fundação Oswaldo Cruz. Coordinadora, Dicionário de favelas Marielle Franco, Rio de Janeiro, Brasil. prof.soniafleury@gmail.com Fundação Oswaldo Cruz Centro de Estudos Estratégicos Fundação Oswaldo Cruz Rio de Janeiro Brazil prof.soniafleury@gmail.com

**Keywords:** Personas en Situación de Calle, Políticas Públicas, Estado, Brasil, People Experiencing Homelessness, Public Policy, State, Brazil

## Abstract

El incremento de la población en situación de calle se ha convertido en una de las contradicciones sociales contemporáneas. En Brasil, este crecimiento persiste incluso frente a resultados positivos de las políticas económicas y asistenciales dirigidas a la reducción de la pobreza, poniendo de relieve la multicausalidad del fenómeno. El ensayo reconstruye la historicidad de los dispositivos de normalización, gubernamentalidad y contraderecho, mostrando cómo la vida desnuda se actualiza en territorios donde el estado de excepción opera como regla. Sobre esta base, analiza las múltiples dimensiones -económicas, políticas, raciales, de seguridad y de cuidado- que convergen en el incremento de la población en situación de calle en Brasil, enfatizando la producción estructural de expropiación, precarización y subjetividades vulneradas; destacando, a su vez, la potencia creativa y política de favelas y periferias, la emergencia de ciudadanías insurgentes y las formas de autogobierno territorial que tensionan el orden neoliberal. Finalmente, propone pensar el cuidado, la solidaridad y lo común como horizontes de transformación capaces de reorientar el Estado hacia un modelo pedagógico y dialógico, fundado en la radical democratización y en la reconstrucción de vínculos sociales basados en la justicia, la autonomía y el respeto a los derechos humanos.

## Abstract (en)

The rise in the population experiencing homelessness has become one of the defining social contradictions of our time. In Brazil, this increase persists even in the face of positive outcomes from economic and social assistance policies aimed at reducing poverty, underscoring the phenomenon’s multicausal nature. The essay reconstructs the historicity of the dispositifs of normalization, governmentality, and counter-right, showing how bare life is actualized in territories where the state of exception operates as rule. Building on this foundation, it analyzes the multiple dimensions - economic, political, racial, security-related, and care-related - that converge in the growth of homelessness in Brazil, emphasizing the structural production of expropriation, precarization, and wounded subjectivities; while also highlighting the creative and political vitality of favelas and peripheral territories, the emergence of insurgent citizenships, and forms of territorial self-governance that challenge the neoliberal order. Finally, it proposes care, solidarity, and the commons as transformative horizons capable of reorienting the State toward a pedagogical and dialogical model grounded in radical democratization and in the reconstruction of social bonds based on justice, autonomy, and respect for human rights.

## Introducción

*Hay en la abyección una de esas violentas y oscuras rebeliones del ser contra aquello que lo amenaza y que le parece venir de un afuera o de un adentro exorbitante, arrojado más allá de lo posible, de lo tolerable, de lo pensable.* Julia Kristeva^1^

Al adentrarme en este tema me invadió una imagen de mi infancia en el campo. Estaba dentro de la camioneta de mi padre, en un camino oscuro y lluvioso, y pude ver grupos de personas, de diferentes edades, cubiertas con mantas que las protegían de la lluvia y ocultaban sus cuerpos, que avanzaban tocando una campana que anunciaba su paso por aquellos parajes. Eran leprosos, sin lugar en el mundo, que necesitaban hacer visible su presencia, a pesar del miedo que provocaban en niñas como yo y de la pena o el rechazo de los adultos. 

Esa experiencia, que quedó grabada en la memoria de una niña, ilustra un momento histórico en el cual la sociedad rechazaba la presencia de personas con esa enfermedad, ocultándolas en leprosarios junto con sus descendientes, respaldada por una pseudociencia que legitimaba la política pública de exclusión de aquello que debía mantenerse oculto, contenido y aislado de la vida social. El (des)encuentro propiciado por la campana que suena, anunciando las presencias indeseables que deberían permanecer ocultadas, opera una revelación y un intercambio de afectos: miedo y asco de un lado, humillación y vergüenza del otro^2^.

Para Foucault^3^, en todas las sociedades se invirtió en el cuerpo dentro de la trama de poderes, pero entre los siglos XVI y XVIII hubo un cambio en la tecnología de poder, con las disciplinas que, a través del control del tiempo y del espacio, pasaron a ejercer un control minucioso de las operaciones de los cuerpos, volviéndolos dóciles y aumentando la eficacia de sus movimientos.

“La penalidad perpetua que atraviesa todos los puntos y controla todos los instantes de las instituciones disciplinarias compara, diferencia, jerarquiza, homogeniza, excluye. En una palabra, normaliza”.^3^

La normalización separa de forma binaria lo normal y lo patológico, pero adquiere distintas modalidades y dispositivos que Foucault^4^ identifica a partir de la historia de las enfermedades. Mientras los leprosos son rechazados, aislados y cercados, la tecnología que emerge del control de la peste es disciplinaria: son mecanismos de poder dispuestos en torno de lo anormal. Al analizar el dispositivo que surge con la vacunación en el combate a la viruela, Foucault afirma que ya no se trata de demarcar y separar enfermos de no enfermos, o de delimitar el territorio, sino de tomar el control de toda la población y permitir su circulación en el territorio. El gobierno de las poblaciones como fuerza productiva modifica la economía del poder, introduce las nociones de riesgo y vulnerabilidad y tiene como mecanismos esenciales los dispositivos de seguridad, modalidad de ejercicio del poder, que el autor denominó gubernamentalidad.

“Por esta palabra, *gubernamentalidad*, entiendo el conjunto constituido por las instituciones, los procedimientos, análisis y reflexiones, los cálculos y las tácticas que permiten ejercer esa forma muy específica, aunque muy compleja, de poder que tiene por objetivo principal a la población, por principal forma de saber la economía política y por instrumento técnico esencial los dispositivos de seguridad”.^4^

Compatibilizar un orden basado en la noción de igualdad jurídica y política en el Estado moderno y la persistencia de asimetrías y exclusiones es tarea de las disciplinas que, para Foucault^3^, constituyen el subsuelo de las libertades formales y jurídicas, una especie de “contraderecho”^5^. En otros términos, la postulación de la igualdad como fundamento de la legitimación del poder político -que es siempre un constructo político y no una consecuencia natural- requiere del gobierno de la población o *gubernamentalidad*, que permite prever los comportamientos y actitudes de los individuos. Para ello, la razón de Estado debe intervenir en la conciencia de las personas, gobernar sus mentalidades, modificar sus opiniones y su modo de actuar, su comportamiento como sujetos económicos y como sujetos políticos^4^.

La pretensión de gobernar las mentalidades de las poblaciones -que le aseguraría prever comportamientos y actitudes y la libre circulación de cuerpos dóciles igualados en la condición político-jurídica hipotética de la ciudadanía^6^, a través de los derechos garantizados por el Estado- no se aplica de manera homogénea, en la medida en que el contraderecho le es constitutivo. Para Agamben^7^, esa condición abre la posibilidad del “estado de excepción”, un territorio fuera del ordenamiento jurídico que, al excluir el campo de sí, este se vuelve incluido como condición permanente^8^.

Paulo Lima^9^ recurre al pensamiento de Agamben para comprender la relación entre el disciplinamiento del cuerpo individualizado y las biopolíticas sobre el cuerpo social, tomando el concepto de *vida desnuda* como la articulación que divide y conjuga vida biológica y vida social; *vida desnuda* entendida “como el primer contenido del poder soberano, que divide y conecta en el cuerpo *bios* político y *zoé* animal, el umbral entre una vida políticamente calificada y la animalización de lo humano”^9^. Ferreira^10^ retoma a Agamben para afirmar que la máquina antropológica produce el reconocimiento humano; sin embargo, contiene en su centro una zona de indiferencia en la cual el producto final no es ni una vida animal ni una vida humana, sino tan solo una vida separada y excluida de sí misma: la vida desnuda: “aquella que cualquiera puede quitar sin cometer homicidio o aquella que cualquiera puede llevar a la muerte, a pesar de ser insacrificable”^10^.

En el interior de esta trama de relaciones de poder es donde queremos situar a la población en situación de calle, como un campo en el cual la vida desnuda se manifiesta, el estado de excepción es norma, el contraderecho impera y las vidas al azar pueden ser eliminadas porque reflejan la imagen en la cual la sociedad no quiere verse. Sin embargo, la exclusión es relacional y no puede comprenderse fuera de la noción de aquello de lo que es apartada, es decir, lo incluido.

Lima^9^ habla de las condiciones de vida de los trabajadores, de la vampirización de su vitalidad en las horas en las que se desplazan hacia el trabajo por las vías del tren, y de la vida dócil que les fue reservada, de los cuerpos negros amenazadores que deben ser controlados en las favelas y periferias. La población trabajadora vive acosada entre la búsqueda de la supervivencia y los temores de perder la capacidad de sostenerla, con la perspectiva siempre presente de cruzar la línea divisoria que separa la inclusión en la condición de sobreviviente de la exclusión propia de la población en situación de calle. Lima concluye que, frente a estas condiciones de vida, el sobreviviente se afirma en el cúmulo de cuerpos que cayeron a su lado. El autor propone que pensemos dónde habitan las potencias-del-no, capaces de producir cortocircuitos en el poder, salirse de los rieles y crear senderos, formas colectivas de resistencia, pequeños gestos de desobediencia, las rebeliones de toda índole.

Nos interesa abordar lo social a partir de su emergencia como “cuestión social”, es decir, como el reconocimiento de nuevos problemas que emergen en la arena política a partir de la transformación de necesidades en demandas, proceso que solo puede realizarse de manera concomitante con la propia construcción de nuevos sujetos políticos. Por lo tanto, la cuestión social pasa a ser reconocida cuando es politizada por nuevos actores que, mediante la construcción de sus identidades, y la formulación de proyectos y estrategias, recolocan la problemática de la integración y de la necesidad de recrear los vínculos sociales.

En este sentido, la emergencia de la cuestión social es siempre un analizador, en el sentido analítico-institucional usado por Lourau^11^, porque revela las contradicciones sociales y expone las fragilidades de la construcción política de una comunidad cohesionada. Hoy, la violencia urbana y la población en situación de calle son las principales cuestiones sociales que exponen las inseguridades y fracturas que imposibilitan sostener el imaginario de una sociedad cuya primacía de la justicia social ordenaría las relaciones económicas, culturales y políticas, asegurando la cohesión social.

A diferencia de los autores europeos, cuyo enfoque subraya la inexorabilidad de las relaciones de poder y dominación sobre la población, la literatura del Sur Global, sin desconocer los rieles de los procesos de subalternización y exclusión, encuentra en las favelas y periferias una efervescencia y una potencia creativa que desafían el orden, crean senderos por fuera de los carriles^9^, reivindican una ciudadanía insurgente^12^, exponen contradicciones y organizan resistencias^13^, producen conocimientos y gestión territorial en las emergencias^14^, e imponen su potencia sobre el terreno de las carencias^15^. Frente a la mercantilización de la vida, del trabajo y de la protección social, surgen colectivos de jóvenes -muchos de ellos egresados de universidades gracias al sistema de cupos-, se crean espacios de constitución identitaria, organizaciones y sujetos políticos, oponiendo la solidaridad a la competencia y al individualismo, en la búsqueda de una estrategia de construcción de lo común^16^ como forma de enfrentar las desigualdades y la destrucción de la sociabilidad impuestas en la fase actual del capitalismo.

## Los carriles: pobreza, desigualdad y calle

Los estudios sobre la población en situación de calle señalan la enorme complejidad en relación con las causas y consecuencias que se acumulan y, muchas veces, se entrelazan. Aun así, es imposible disociar el aumento de la población en situación de calle de la condición de pobreza, en un país en el cual, según datos preliminares del Censo 2022 divulgados por el Instituto Brasileiro de Geografia e Estatistica, más de un tercio (35%) de los trabajadores recibe hasta un salario mínimo^8^^,^^17^, y solo el 7,6% recibe por encima de cinco salarios mínimos. A pesar de la reciente recomposición del valor del salario mínimo, Brasil poseía, en 2022, el segundo peor salario mínimo de la lista de 31 países de la Organización para la Cooperación y el Desarrollo Económicos (OCDE)^18^.

Los especialistas ven, en el gobierno actual, la revalorización del salario mínimo, los aumentos reales de los ingresos, la reducción de la subocupación y del desánimo, y la caída del desempleo como evidencias de un cuadro de resiliencia^19^ frente al escenario macroeconómico actual, marcado por una deuda y tasas de interés elevadas. Incluso con el leve retroceso de la tasa de informalidad, 39,5 millones de los 103 millones de trabajadores trabajaban sin contrato formal ni registro en el *Cadastro Nacional de Pessoas Jurídicas* (CNPJ), según datos de la *Pesquisa Nacional por Amostra de Domicílios* **(**PNAD) divulgados en septiembre pasado^20^.

Las políticas públicas de recuperación del valor del salario mínimo y de aumento del empleo afectan a la totalidad del mercado de trabajo, así como a los beneficiarios de, por ejemplo, el Beneficio de Provisión Continua (BPC), que es una pensión no contributiva, y las jubilaciones, constitucionalmente vinculados al valor del salario mínimo. Sin embargo, siempre pesa la amenaza de la desvinculación de los beneficios en favor del ajuste fiscal y de la producción de superávit, mientras el Congreso se niegue a diseñar medidas fiscales para gravar las grandes fortunas.

Las políticas económicas, asistenciales y de promoción de la seguridad alimentaria tuvieron un impacto positivo en la vida de 2,2 millones de hogares que dejaron de presentar algún grado de inseguridad alimentaria en 2024. Sin embargo, la inseguridad alimentaria continúa reflejando las persistentes desigualdades, y presenta mayores proporciones en las regiones Norte y Nordeste, en los hogares de las áreas rurales, en los hogares encabezados por mujeres jefas de hogar y por personas pardas, y en aquellos cuyos responsables tienen hasta la educación primaria completa.

A pesar del avance notable en la lucha contra el hambre, lo que permitió que Brasil en 2024 tenga el menor índice de inseguridad alimentaria de su historia, es alarmante la constatación^21^, basada en los datos del módulo de Seguridad Alimentaria de la PNAD que, en 2024:

“Dos de cada tres (66,1%) domicilios con ingreso per cápita de hasta un salario mínimo se encontraban en inseguridad alimentaria. Esta proporción sube a 71,9% entre los hogares de hasta un salario mínimo per cápita en el grado moderado o grave de inseguridad alimentaria”.^21^

Y esto sucede incluso en los hogares cuyo responsable estaba trabajando, el 20,7% sufrió algún nivel de inseguridad alimentaria^21^.

La informalidad, además de impedir el acceso de los trabajadores a los derechos laborales, presenta una trayectoria laboral discontinua, lo que contribuye a vulnerabilizar a ese segmento de la población, sometiéndolo a constantes riesgos de inseguridad alimentaria y pérdida de la capacidad de mantener el domicilio. Dentro del contingente de población que se encuentra en la informalidad, se destacan las trabajadoras domésticas, un grupo de seis millones en 2024, cuya precariedad se expresa, según el Instituto Doméstica Legal, en el hecho de que el 70% de las trabajadoras domésticas continúa en la informalidad y tienen un salario promedio por debajo del salario mínimo en el período^22^. A esta situación de precarización e inseguridad se suma el hecho de que más de la mitad de las familias encabezadas por mujeres con hijos viven por debajo de la línea de pobreza, siendo las mujeres negras el 64,4% de las madres solas en situación de pobreza^23^.

A pesar de las tendencias de reducción de la tasa de desempleo y del porcentaje de personas que viven en pobreza extrema, y de que Brasil haya salido del Mapa del Hambre de la *Food and Agriculture Organization* (FAO), en parte debido al aumento de la cobertura del Programa Bolsa Família -que hoy alcanza a 20 millones de familias-, la población en situación de calle viene creciendo, alcanzando casi 350 mil personas en 2025 según los registros de inscritos en el Cadastro Único^24^, que da cobertura de la población más vulnerable. Tal expansión de la población en situación de calle ya venía siendo detectada desde la pandemia de covid-19 por Natalino^25^, cuyo número se triplicó entre 2020 y 2023.

La discrepancia entre los datos que muestran los resultados positivos de las políticas económicas y asistenciales dirigidas a la reducción de la pobreza y el aumento de la población en situación de calle nos llevan a reflexionar acerca de la multicausalidad del fenómeno, exigiendo pensar las inseguridades que asolan a la población más allá de explicaciones simplistas. Resulta evidente que el sostenido aumento de la población en situación de calle no puede explicarse como mero reflejo de la economía de la pobreza y de la asistencia a los pobres. Es imprescindible considerar un conjunto multicausal que incrementa las inseguridades socioeconómicas y los conflictos intergrupales e intrafamiliares.

La generación de una “sobrepoblación relativa” como consecuencia de la acumulación capitalista fue establecida por Marx como un rasgo peculiar del modo de producción capitalista, en el cual “la población obrera, pues, con la acumulación del capital producida por ella misma, produce en volumen creciente los medios que permiten convertirla en relativamente supernumerária”^26^. A diferencia de su postulada funcionalidad como ejército industrial de reserva, José Nun^27^ distingue entre la fracción de la sobrepoblación que mantiene relación con el sector productivo y aquella población sobrante que denominó *masa marginal*, que no cumple con esa funcionalidad. Al realizar esta diferenciación teórica, la contribución de Nun conserva su total actualidad con foco en la realidad latinoamericana, y puede reconocerse en el uso contemporáneo del término *precariado*^28^ para designar a la fracción de la población que está siendo cada vez más expulsada del mercado de trabajo. Los *descartables* constituyen un fenómeno que forma parte del proceso de precarización y flexibilización de las relaciones y derechos laborales, así como de las profundas transformaciones ocurridas en la era de la globalización, la liberalización y la reestructuración productiva. En consecuencia, la inseguridad permanente, el miedo a la exclusión, la falta de perspectivas de futuro y la competencia por la supervivencia asolan a la población trabajadora, cada vez más fragmentada.

Según el Instituto Brasileiro de Geografia e Estatistica^29^, hubo un aumento del 25,4% de trabajadores de aplicaciones entre 2022 y 2024, quienes trabajan más horas por semana que los trabajadores no plataformizados, y perciben un ingreso por hora 8,3% inferior al de estos últimos.

“Mientras el 43,8% de los no plataformizados se encontraba en la informalidad, entre los trabajadores plataformizados ese porcentaje era del 71,1%. Además, los trabajadores plataformizados contaban con menos personas que contribuyen a la previsión social (35,9% vs 61,9%)”.^29^

A pesar de trabajar más y ganar menos, los trabajadores plataformizados viven en una inseguridad permanente, como resultado de las precarias condiciones de trabajo y de la ausencia de políticas de cobertura para las situaciones de riesgo que enfrentan.

Sin embargo, el trabajo es un factor crucial al afectar el bienestar y moldear identidades y comunidades^30^. La condición de desempleo y, añadimos, la de subempleo, además de privar a las personas de derechos laborales y sociales, las sitúa en una zona de vulnerabilidad que reduce la autoconfianza y la autoestima, con implicaciones en el auto y heterorrespeto, en las condiciones de bienestar y en la salud física y mental de la persona y de su núcleo familiar. Ello tiene consecuencias en las relaciones de reconocimiento^31^, y puede implicar, además de la privación de derechos y la exclusión, situaciones de irrespeto, malos tratos y violaciones, degradación y ofensa.

Si, por un lado, es imprescindible considerar la especificidad de la vida de la población en situación de calle, por otro lado, no puede desconocerse su origen de clase, cuya reproducción, cada vez más ampliada, forma parte de la acumulación capitalista.

La financiarización de la economía, asociada a la globalización y al avance de nuevas tecnologías productivas, agudiza este cuadro, al subordinar todas las relaciones a la lógica de la acumulación financiera, aumentando la concentración de ingresos en la primera década de este siglo a niveles que retroceden al inicio del siglo pasado. Es decir, retroceden al período previo a los años dorados, cuando las políticas públicas, fundadas en los principios de crecimiento con solidaridad y justicia social, lograron reducir significativamente las desigualdades en los países más desarrollados^32^. Los mercados financieros, por un lado, además de no producir riqueza, no incorporan empresas productivas ni trabajadores, impulsando un proceso de desindustrialización. Por otro, dada su mayor movilidad espacial, conducen a la concentración del ingreso mediante la sustitución de estructuras progresivas de tributación por estructuras regresivas, que penalizan más a la población de menores ingresos y estrechan las bases tributarias, inviabilizando las políticas distributivas de protección social, amenazando así la cohesión social^32^.

Streeck^33^ concluye que los años dorados del capitalismo democrático -desde la posguerra hasta las décadas de 1960 y 1970- representan una conjunción rara entre crecimiento económico y distribución. A partir de entonces, creció la oposición entre el “pueblo del mercado” y el “pueblo del Estado” (este último incluye a ciudadanos que dependen de las políticas públicas distributivas amenazadas y de las restricciones al Estado de bienestar). Políticas inflacionarias, aumento del endeudamiento privado y público, políticas de austeridad y de liberalización financiera fueron implementadas frente a ciclos de crisis económica cada vez más acelerados. Los Estados, que antes eran recaudadores, se transformaron en grandes deudores, incapaces de afrontar las demandas económicas y sociales mediante políticas públicas.

Tomemos el caso de la actual política macroeconómica brasileña, en la que al mantener en 15% la tasa Selic (tasa de interés de referencia de la economía brasileña), el país destinó cerca de un billón de reales en 2024 al pago de la deuda pública federal^34^, alimentando el parasitismo rentista y aumentando la desigualdad en el país. La financiarización de la economía reduce los recursos destinados a las políticas públicas y eleva el nivel de endeudamiento de los trabajadores.

“Según (datos del) Banco Central, incluso sin captar los datos de endeudamientos fuera del sistema financiero, como créditos en tiendas, luz y agua, (el endeudamiento) alcanzó el 48,7% del ingreso disponible acumulado en los últimos doce meses de enero de 2025, uno de los niveles más altos de la serie histórica”.^35^

El aumento exponencial de las desigualdades, por un lado, y de la inseguridad, por el otro, empuja muchas veces a la población endeudada a la situación de calle. La pérdida de ingresos y de estabilidad son factores cruciales en la desorganización de las relaciones sociales, llevando a una parte de la población a la condición de estar en la calle. No obstante, otros factores no económicos también deben ser considerados.

La política de guerra contra las drogas fracasó completamente, alimentando la violencia y el dominio de los territorios de favelas y periferias por milicias armadas y narcotraficantes. La autorización de las fuerzas de seguridad y represión para ejecutar operaciones de guerra en esos territorios ha sido ineficaz desde el punto de vista del rescate del poder estatal, causando enormes daños a la población residente, que vive en condiciones de inseguridad frente a la creciente violencia estatal y la violencia delictiva. Se trata de una negación de los derechos humanos y sociales autorizada por el Estado, una verdadera licencia para matar jóvenes negros favelados, aquello que Mbembe denominó necropolítica^36^.

Estefanía Ciro^37^, especialista en economía de las drogas, argumenta que la falta de regulación promueve la regulación armada, en lugar de promover la prevención y tratamientos adecuados como cuestión de salud pública. Por medio del Argumento de Incumplimiento del Precepto Fundamental (ADPF nº 635) de las favelas^38^, el Supremo Tribunal Federal buscó frenar las graves violaciones a los preceptos constitucionales derivadas de la política de seguridad pública del estado de Río de Janeiro, evidenciadas en la creciente letalidad de la actuación policial en las favelas. Aun así, el mundo asistió, consternado, a la masacre promovida por el gobierno de Río de Janeiro, con la muerte de más de 120 personas en las favelas de los complejos de la Penha y del Alemão, abandonando más de 70 cadáveres en el monte, donde fueron ejecutados.

En 2018, Naciones Unidas lanzó un documento^39^ en el cual se establecen directrices internacionales sobre derechos humanos y políticas de drogas, que representaron un giro en el tratamiento dado a la guerra contra las drogas. Según el Alto Comisionado de la ONU^40^, esa guerra destruyó innumerables vidas y perjudicó comunidades enteras y señala como consecuencias perjudiciales “tasas de encarcelamiento masivo, aumento de crímenes y violencia relacionados con drogas, cifras récord de muertes relacionadas con drogas, aumento de la producción ilegal de drogas y estigmatización y discriminación de comunidades enteras” ^(^^40^.

La violencia urbana ha sido uno de los principales factores de aumento de la inseguridad de la población, emergiendo como una modalidad de estructuración de las relaciones y acciones sociales abarcada por el concepto de *sociabilidad violenta*^41^. Tal modalidad no se restringe a los territorios periféricos y/o en conflicto, sino que atraviesa toda la sociedad, que comienza a buscar la resolución de conflictos mediante la fuerza y la coacción, o incluso mediante la eliminación del otro, cuya alteridad se vive como amenaza, como lo demuestran el aumento de casos de intolerancia religiosa, los altos índices de homicidio y, en especial, el creciente número de feminicidios.

La ausencia de políticas habitacionales, de urbanización de favelas y de movilidad urbana, con capacidad para responder de manera efectiva a las demandas de más de 16 millones de personas que residen en las 12.348 favelas y comunidades urbanas, según el Censo de 2022, precariza aún más las condiciones de vida de esa población, además de someterla a riesgos y emergencias climáticas. Vivir en condiciones de degradación, conviviendo con basura y zanjas de desagüe, como resultado de la ausencia de políticas públicas efectivas, convierte la vulnerabilidad y la inseguridad en estrategias políticas de subalternización de una población mayoritariamente compuesta por personas pardas y negras.

Fraser^42^ argumenta que la expropiación es, de hecho, constitutiva de la sociedad capitalista y de su entrelazamiento con el racismo y el patrimonialismo. El Estado constituye al trabajador libre, explotado en la condición de asalariado, pero conserva los beneficios de la ciudadanía, mientras que el trabajador expropiado está sujeto a una dominación no mediada por el contrato de trabajo, por lo que es incapaz de establecer límites e invocar protección. La condición de expropiación, posición inferior en la jerarquía material y simbólica, no se reduce al nivel económico, pues la sujeción requiere ser comprendida en su dimensión política, en la cual el patrimonialismo y la racialización operan como formas de sujeción y subjetivación de grupos poblacionales y territorios. Es necesario entender cómo la fragmentación de los trabajadores entre explotados y expropiados tiene consecuencias más allá de la materialidad, y construyendo subjetividades y reproduciendo dinámicas políticas de subalternización.

## La senda: el cuidado, la solidaridad, lo común

Al estudiar las paradojas de la modernización en América Latina, Lechner^43^ toma el ejemplo chileno, en un período de crecimiento económico en el cual el producto bruto interno bruto (PBI) se mantuvo por encima del 7% anual, para mostrar que la modernización va más allá del ámbito económico y afecta al conjunto de la sociedad, y concluir que los avances económicos no guardaron relación con la subjetividad de las personas. “La subjetividad importa”, enseña Lechner^43^, al identificar el profundo malestar de la población chilena en ese período, basado en ciertas inseguridades que le permitieron identificar tres tipos de miedo:

“el miedo al Otro, que suele ser visto como potencial agresor; el miedo a la exclusión económica y social; el miedo al sinsentido a raíz de una situación social que parece estar fuera de control”.^43^

El autor advierte que los miedos son fuerzas peligrosas que pueden provocar reacciones agresivas y de odio y son presas fáciles de la manipulación, fenómeno que estamos presenciando con la ola de populismos autoritarios en el mundo actual. Esto nos lleva a preguntarnos cuánta desigualdad es capaz de soportar el régimen democrático e incluso el capitalismo, así como a pensar en la construcción de alternativas transformadoras.

El conflicto redistributivo vuelve a escena en la región latinoamericana en la segunda década de este siglo, en una coyuntura económica desfavorable para la exportación de productos primarios, generando manifestaciones populares, amenazas de golpes y el retorno de gobiernos populistas autoritarios, que no respetan los derechos humanos y amenazan a los grupos más vulnerables, vistos como blancos a ser subordinados o eliminados. El desmantelamiento de las políticas de protección social^44^ es parte constitutiva de la desdemocratización que vivimos recientemente en Brasil y que prosigue en países vecinos.

El ocaso del progresismo ocurre por su incapacidad para construir un modelo económico ecológicamente sostenible, capaz de atender las demandas de incorporación tecnológica soberana y de justicia social. Políticamente, buscó construir alianzas pragmáticas con élites políticas y económicas conservadoras y tradicionales, además de mantener esquemas de negociación de cargos públicos y prebendas, impidiendo la renovación de las prácticas políticas y una participación efectiva de las fuerzas populares en la distribución del poder. En el plano cultural, se observa la ausencia de un proyecto político-ideológico emancipador, que involucre a los sectores populares en un proceso de constitución de nuevas subjetividades de base solidaria, condición para abrir camino a superar la subjetividad individualista y competitiva construida por el neoliberalismo bajo la aureola del emprendimiento, que termina culpabilizando a las víctimas por su fracaso.

Un proyecto de cambio basado en lo común requiere la construcción de actores colectivos y una base sólida para impulsar transformaciones estructurales en la sociedad. En cambio, mediante las políticas de transferencia implementadas por el progresismo, se buscó crear consumidores, en lugar de invertir en la construcción de una ciudadanía emancipadora, lo que terminó resultando en la frustración de las expectativas de ascenso social, estabilidad y seguridad respecto al futuro. La individualización de los riesgos elimina el fundamento de la condición común de la inseguridad, socavando las posibilidades de construir alternativas colectivas frente a los miedos y resentimientos que terminan siendo apropiados por los llamamientos simplistas de los partidos de derecha.

Las décadas de políticas de austeridad y debilitamiento de las capacidades estatales fueron acompañadas por la sobrevaloración de la lógica y la ética del mercado, donde el individualismo, la competencia y el consumismo se convierten en valores centrales para la organización de las relaciones económicas y sociales. La difusión de la ideología del emprendimiento organiza esa subjetividad en la cual se ancla la hegemonía neoliberal, contaminando incluso las políticas públicas. El mercado capturó las nociones de autonomía, libertad e innovación, lo que fue reforzado por los cambios tecnológicos y las redes sociales. Sin embargo, estos son valores básicos de los individuos, que deberían ser parte constitutiva de un proyecto emancipador.

Un gran desafío consiste en cómo actualizar las agendas que defienden la justicia y la inclusión social, más allá de las políticas tradicionales de protección social -que son imprescindibles, pero que se sitúan en el reino de las necesidades- cuando los jóvenes se movilizan hoy a partir de la autonomía y el deseo. En este sentido, es crucial la distinción formulada por el filósofo Alain Renaut^45^ entre autonomía e independencia, según la cual

“…la libertad en tanto autonomía parte del presupuesto de la existencia de una humanidad común, irreductible a la afirmación de cada individualidad, y a la cual cada individuo debe someterse. Muy distinto sería el ideal de independencia, en el que se enfatizan las libertades individuales, la preocupación por sí mismo, el culto a las felicidades particulares y la deserción del espacio público”.^45^

Para Ribeiro^46^, la independencia estaría asociada a un individualismo extremo, algo semejante a la posición defendida por los neoliberales.

La pandemia exacerbó las contradicciones y limitaciones del progresismo al intensificar las desigualdades y revelar su incapacidad para formular políticas sanitarias adecuadas al cuidado de la población que vive en las favelas y periferias o en situación de calle. Las innumerables iniciativas de autoorganización de la población favelada para hacer frente a la pandemia^15^, ante la ausencia de políticas públicas adecuadas a las condiciones de trabajo informal, el insuficiente acceso a condiciones sanitarias y a servicios como salud e Internet, entre otras carencias, demostraron, por otro lado, la enorme potencia existente en esos territorios, en especial en aquellos cuya sociabilidad y luchas han logrado construir una identidad fuerte y una red de relaciones y organizaciones colectivas.

Más allá de la coyuntura crítica de la pandemia, Fraser^42^ identifica la crisis del cuidado como consecuencia de la canibalización actualmente producida por el capitalismo, en la medida en que el ansia de acumulación ilimitada amenaza con desestabilizar las propias capacidades y procesos reproductivos de los cuales el capital depende. No obstante, consideramos que el cuidado debe ser tomado como el lugar de posibilidad de las luchas contrahegemónicas, pues se constituye en un encuentro de alteridades, un coloquio singular que puede tanto rectificar las relaciones de subalternización como introducir las semillas de la emancipación. Si el cuidado involucra una dimensión técnica, también implica afectos y una dimensión moral. La aceptación del otro no es una cuestión técnica, sino un valor basado en la solidaridad, la justicia social y el respeto a los derechos humanos. Condiciones que solo tienen vigencia en la radical subordinación de las políticas públicas a la primacía de la justicia social, principio que se opone a la mercantilización de la protección social, orientada por la búsqueda de la rentabilidad y excluyendo a quienes no poseen capacidad financiera.

La negación de derechos a ciertos grupos sociales exacerba la convivencia paradójica entre un orden jurídico y político basado en el principio de igualdad básica entre los ciudadanos y la preservación de niveles exponenciales de desigualdad y exclusión económica, cultural y social, como base de la superexplotación sostenida en el sexismo y el racismo, entre otras formas de discriminación. Holston^47^ identifica la emergencia de ciudadanías insurgentes, en especial entre quienes residen en los márgenes de las grandes metrópolis, como consecuencia de la existencia de disyunciones en las democracias que no logran garantizar el conjunto de derechos de ciudadanía a todos sus habitantes.

En estas situaciones, las organizaciones territoriales que reivindican mejoras y derechos relativos a su vida y bienestar cotidianos politizan su día a día y se organizan de forma asociativa en torno a estas banderas. Mientras la ciudadanía formal se sitúa en la esfera pública, quienes de ella están excluidos se organizan en torno a la vivienda, al espacio privado, al territorio, en suma, a la esfera reproductiva, desde la cual formulan sus demandas en términos de derechos. Se trata, por lo tanto, de agenciamientos complejos, que abarcan micro y macropolíticas, esferas pública y privada. Según el concepto creado por Gilles Deleuze y Félix Guattari^48^, todo agenciamiento es, en primer lugar, territorial^48^. Si en el territorio se crea el agenciamiento, quienes actúan crean relaciones, conexiones en red, lenguaje y significaciones.

El *Movimento Nacional da População de Rua* (MNPR), lanzado en 2005 y articulado a nivel nacional, es un ejemplo de cómo la insurgencia puede transformarse en políticas públicas, constituyéndose como un actor político que pasó a incidir y convertirse en socio en las políticas del Sistema Único de Asistencia Social (SUAS) y del Sistema Único de Salud (SUS), participando también en la definición e implementación de la *Política Nacional da População de Rua* (Decreto 7053/2009). Esa trayectoria de politización de la población en situación de calle culmina con su reconocimiento como actor político con la audiencia pública del Supremo Tribunal Federal, en la Argumento de Incumplimiento de Precepto Fundamental (ADPF 976), convirtiendo la vida en la calle en una cuestión social^49^. La militancia en este movimiento favorece la construcción de redes de apoyo que incrementan la autoestima, la capacidad de agencia y la positivación de la identidad.

Lo común implica la construcción de una subjetividad colectiva y solidaria, frecuentemente emanada de acciones colectivas de autogobierno, pero también requiere su institucionalización mediante políticas e instituciones públicas gubernamentales, que trasciendan la dimensión del autogobierno local. Asumiendo una dirección de influencia mutua, el fortalecimiento de lo común demandará un nuevo modo de gobernar, en el cual los procesos decisorios incorporen elementos de democracia deliberativa y la gestión en redes de políticas^50^, de forma más horizontal y consensuada, involucrando a los diferentes actores implicados.

La construcción de lo común requiere la publicización de los espacios, con la desmercantilización de la apropiación privada lucrativa, posibilitando formas autogestionarias de definición de reglas y de actuación colectiva en la esfera de la producción en formas de economía solidaria^51^^,^^52^, innovaciones en la planificación y gestión del espacio urbano^53^, y el reconocimiento de las innumerables manifestaciones colectivas en la esfera reproductiva, ya sean en el campo cultural o en el aprovisionamiento de bienes y servicios.

Tenemos ante nosotros el desafío de repensar la protección social en términos de un paradigma de sociabilidad que recupere los valores de emancipación, autonomía y justicia social, articulando las áreas tradicionalmente conocidas de las políticas sociales con las grandes cuestiones del trabajo, la vivienda, el medio ambiente, la violencia y la política económica. A las políticas públicas de protección social será necesario incorporar la potencia insurgente generada en los territorios, reposicionando, dentro de un paradigma solidario, la idea de autonomía, que involucra el deseo de los individuos en relación con sus capacidades creativas e innovadoras, fuertemente presentes en la sociedad actual. Frente al miedo y al resentimiento, nos vemos compelidos a ofrecer esperanza: un proyecto común de emancipación y transformación social.

Fundamentalmente, transformar la sociedad en dirección a lo común es transformar el Estado hacia su radical democratización y su capacidad efectiva de ejercer un modelo dialógico de interacción con la sociedad, que he denominado un Estado pedagógico, rescatando la tradición de Paulo Freire, quien nos alerta sobre la importancia del *esperanzar*: construir juntos un futuro capaz de interpelar a los jóvenes como sujetos colectivos y deseantes, capaces de crear un mundo mejor.

“Es necesario tener esperanza, pero tener esperanza del verbo *esperanzar*; porque hay gente que tiene esperanza del verbo *esperar*. Y esperanza del verbo *esperar* no es esperanza, es espera. *Esperanzar* es levantarse, *esperanzar* es ir atrás, *esperanzar* es construir, *esperanzar* es no desistir. *Esperanzar* es llevar adelante, *esperanzar* es unirse con otros para hacer de otro modo…”.^54^

## References

[1] Kristeva J (1980). Pouvoirs de l’horreur: Essai sur l’abjection.

[2] Rui T (2021). Nojo, humilhação e vergonha no cotidiano de usuários de crack em situação de rua. Anuário Antropológico.

[3] Foucault M (1977). Vigiar e Punir: História da Violência nas prisões.

[4] Foucault M (2008). Segurança, território, população.

[5] Fleury S (2011). Desigualdades injustas: o contradireito à saúde. Psicologia & Sociedade.

[6] Fleury S (2021). Estado sin ciudadanos: seguridad social en América Latina.

[7] Agamben G (2007). Estado de exceção.

[8] Martins L (2015). Estado de Exceção Permanente: o campo e a experiência biopolítica. Sequência (Florianópolis).

[9] Junges M (2025). Vida nos trilhos: corpos sobreviventes e a resistência que brota da periferia brasileira (Entrevista especial com Paulo Ricardo Barbosa de Lima).

[10] IHU, Revista del Instituto Humanitas Unisinos (2007). Agamben e a vida nua: produto final da máquina antropológica.

[11] Lourrau R (1975). El Análise Institucional.

[12] Houston J (2013). Cidadania Insurgente: disjunções da democracia e da modernidade no Brasil.

[13] Fleury S (2018). Capitalismo, democracia, cidadania: Contradições e insurgências. Saúde em Debate.

[14] Faria CP, Ferreira T, Baptista RR, Gracie R, Mota P (2025). Geração cidadã de dados. Dicionário de Favelas Marielle Franco Wikifavelas.

[15] Fleury S, Menezes P (2020). Pandemia nas Favelas: entre carências e potências. Saúde em Debate.

[16] Fleury S, Aguilar PL, Hopp MV, Minteguiaga A, Crojethovic M (2025). Lo común en jaque: Reconfiguraciones de la cuestión social y la política sociolaboral en América Latina.

[17] Catto A (2025). Mais de um terço dos trabalhadores do país recebe até um salário mínimo, diz IBGE. G1.

[18] Zanobia L (2022). Brasil amarga vice-lanterna no ranking do salário mínimo da OCDE. Veja.

[19] Lameiras MAP (2025). Desempenho recente do mercado de trabalho. Carta de Conjuntura.

[20] Abdala V (2025). Informalidade recua no mercado de trabalho em janeiro, diz IBGE. Agencia Brasil.

[21] Renaux P, Pirrho S (2025). Mais de dois milhões de lares saem da insegurança alimentar em 2024. Agência IBGE Notícias.

[22] Martello A (2025). Dia Nacional da Trabalhadora Doméstica: informalidade e precarização são desafios da categoría. G1.

[23] Habitat para a Humanidade (2025). Mães solo no Brasil: a realidade da pobreza e a ajuda da Habitat.

[24] Almeida C, Foletto M (2025). Fora do alcance: população em situação de rua cresce, mesmo com recuo da fome e do desemprego. O Globo.

[25] Natalino MA (2024). A população em situação de rua nos números do cadastro único.

[26] Marx K (1984). O Capital: Crítica da Economia Política. Livro Primeiro: O Processo de Produção do Capital.

[27] Nun J (1969). Superoblación relativa, ejercito industrial de reserva y masa marginal. Revista Latinoamericana de Sociología.

[28] Standing G (2013). O precariado: A nova classe perigosa.

[29] Gomes I, Peters J (2025). Número de trabalhadores por aplicativos cresceu 25,4% entre 2022 e 2024. Agência IBGE Notícias.

[30] Suppa N (2021). Unemployment and subjective well-being. Global Labor Organization.

[31] Honneth A (2003). Luta por reconhecimento: A gramática moral dos conflitos sociais.

[32] Oliveria F (2021). Piketty e as desigualdades no capitalismo: colocando alguns pingos nos is na análise de “O capital no século XXI”. Economia e Sociedade.

[33] Streeck W (2011). The Crisis of Democratic Capitalism. New Left Review.

[34] Kliass P (2025). O rombo de 1 trilhão de reais. outras Palavras.

[35] Lavinas L, Bertran MP (2023). Dívida e endividamento: o novo normal das famílias brasileiras. Boletim Lua Nova.

[36] Mbembe A (2018). Necropolítica: biopoder, soberania, estado de exceção, política da morte.

[37] Ciro E (2025). Por una política anticapitalista sobre drogas. Jacobin.

[38] (2025). Equipe do Dicionário de Favelas Marielle Franco. ADPF 635 - ADPF das Favelas. Dicionário de Favelas Marielle Franco Wikifavelas.

[39] International Centre on Human Rights and Drug Policy, United Nations Human Rights, UNAIDS, World Health Organization (2019). Diretrizes internacionais sobre direitos humanos e política de drogas.

[40] Nações Unidas Brasil (2025). Alto Comissário da ONU: “Guerra às drogas destruiu inúmeras vidas e prejudicou comunidades inteiras”.

[41] Silva LAM (2004). Sociabilidade violenta: por uma interpretação da criminalidade contemporânea no Brasil urbano. Sociedade & Estado.

[42] Fraser N (2024). Capitalismo canibal: como nosso sistema está devorando nossa democracia, o cuidado e o planeta e o que podemos fazer a respeito disso.

[43] Lechner N (1998). Nuestros Miedos. Estudios Sociales, Revista Universitaria Semestral.

[44] Fleury S (2024). Cidadania em perigo: Desmonte das políticas sociais e desdemocratização no Brasil.

[45] Renaut A (1989). A era do indivíduo.

[46] Ribeiro EEM (2024). Liberalismos Identitários. A Terra é Redonda.

[47] Holston J (2008). Insurgent citizenship: Disjunctions of democracy and modernity in Brazil.

[48] Deleuze G, Guatarri F (1997). Mil platôs: capitalismo e esquizofrenia.

[49] Rui D (2025). A população em situação de rua chega ao Supremo Tribunal Federal: o caminho por direitos e reconhecimento a partir de uma audiência pública - a ADPF 976. Saúde e Sociedade.

[50] Fleury S, Ouverney A (2007). Gestão de Redes.

[51] Singer P (2002). Introdução à Economia Solidária.

[52] Rouillé D’Orfeuil H (2002). Finanzas internacionales y solidaridad: hacia una red bancaria mundial y solidaria.

[53] Subirats J (2016). El Poder de lo Próximo: Las virtudes del municipalismo.

[54] Freire P (1993). Pedagogía de la esperanza: un reencuentro con la pedagogía del oprimido.

